# The Impact of Rotating Night Shift Work and Daytime Recharge on Cognitive Performance Among Retired Nurses

**DOI:** 10.3389/fnagi.2021.827772

**Published:** 2022-01-25

**Authors:** Jinghuan Gan, Xiao-Dan Wang, Zhihong Shi, Junliang Yuan, Meiyun Zhang, Shuai Liu, Fei Wang, Yong You, Peifei Jia, Lisha Feng, Junying Xu, Jinhong Zhang, Wenzheng Hu, Zhichao Chen, Yong Ji

**Affiliations:** ^1^Department of Neurology, China National Clinical Research Center for Neurological Diseases, Beijing Tiantan Hospital, Capital Medical University, Beijing, China; ^2^Tianjin Key Laboratory of Cerebrovascular and of Neurodegenerative Diseases, Department of Neurology, Tianjin Huanhu Hospital, Tianjin Dementia Institute, Tianjin, China; ^3^NHC Key Laboratory of Mental Health (Peking University), Department of Neurology, National Clinical Research Center for Mental Disorders (Peking University Sixth Hospital), Peking University Sixth Hospital, Peking University Institute of Mental Health, Beijing, China; ^4^Department of Neurology, Tianjin People’s Hospital, Tianjin, China; ^5^Department of Neurology, Yuncheng Central Hospital of Shanxi Province, Yuncheng, China; ^6^Department of Neurology, Second Affiliated Hospital of Hainan Medical University, Haikou, China; ^7^Department of Neurology, The Second Affiliated Hospital of Baotou Medical College, Baotou, China; ^8^Department of Encephalopathy, Research Institute of Traditional Chinese Medicine, Tianjin University of Traditional Chinese Medicine, Tianjin, China; ^9^Department of Neurology, Tianjin Baodi People’s Hospital, Tianjin, China; ^10^Department of Neurology, Cangzhou People’s Hospital, Cangzhou, China

**Keywords:** shift work, cognitive impairment, dementia, sleep disorders, daytime sleepiness

## Abstract

**Introduction:**

The exact relationship between long-term shift work (SW) and cognitive impairment (CI) has been poorly understood. The effects of the long-term rotating night SW (RNSW) combining daytime recharge (DTR) on cognitive function were investigated.

**Methods:**

A total 920 retired nurses and 656 retired female teachers aged ≥50 years were analyzed. Participants who worked at least once per week for 8 hat night for more than 1 year were defined as the SW group, and those without a regular nighttime shift were defined as the control group. The associations among duration, frequency, and DTR of RNSW, and neuropsychological assessments were ascertained by regression models.

**Results:**

Participants with RNSW had a significantly higher proportion of mild CI (MCI), both amnestic MCI (aMCI) (14.4% in 11–20 years, *p* < 0.05, and 17.8% in > 20 years, *p* < 0.001) and non-amnestic MCI (naMCI) (8.1% in 11–20 years, *p* < 0.05), as well as dementia (1.5% in 1–10 years, and 11.7% in > 20 years, *p* < 0.05) compared to controls (8.4% with aMCI, 4.4% with naMCI, and 7.0% with dementia, respectively). There were significant negative relationships between general times of night SW and scores of Mini-Mental State Examination (MMSE) (*R* squared = 0.01, *p* = 0.0014) and Montreal Cognitive Assessment (MoCA) (*R* squared = 0.01, *p* = 0.0054). Participants with ≥1 h of DTR and ≥ 11 years of RNSW were about 2-fold more likely to experience MCI compared with the subjects in the control group, especially with 3–5 h (odds ratio [OR]: 2.35; 95% confidence interval: 1.49–3.68, *p* < 0.001).

**Conclusion:**

The long-term RNSW was associated with a higher risk of CI, especially aMCI and dementia, and the problem cannot be improved by DTR.

## Introduction

Shift work (SW) has been prevalent in modern society; however, its far-reaching effects on cognitive performance are only beginning to be understood from a scientific perspective (Jørgensen et al., 2020). SW has a wide-ranging impact on circadian and sleep functioning, and it seems likely to share several possible mechanisms, such as sleep disorders that involved in increasing the risk of cognitive impairment (CI). Emerging evidence indicates that SW may increase the risk of developing Alzheimer’s disease (AD), because circadian rhythm disturbances have been shown to interfere with the accumulation and clearance of β-amyloid (Aβ) (Lucey et al., 2017). Thus, sleep and cognition studies are important for developing strategies for better general models of how the cognitive system dynamically adjusts to impairments in processing (Whitney and Hinson, 2010).

Reportedly, >24 h of sleep deprivation can lead to 30–70% decline in cognition, especially in attention (Massar et al., 2019), alertness, sensory perception (Daviaux et al., 2014), learning and memory (Wilckens et al., 2018), and executive function (De Almondes et al., 2020). Thus, the extent to which sleep disorders affect a particular cognitive process may depend on several factors, including the magnitude of the global decline in general alertness and attention, the degree to which the specific cognitive function depends on emotion-processing networks, and the extent to which cognitive process can draw upon associated cortical regions for compensatory support. It is evident that some aspects of higher-level cognitive capacities remain degraded by sleep disorders despite the restoration of alertness and vigilance with stimulant countermeasures, suggesting that sleep loss may affect specific cognitive systems in addition to the effects produced by global cognitive declines or impaired attentional processes.

As age increases, the negative effects of sleep disorders due to SW on CI also increase; however, the exact relationship between age, SW, and cognitive function is debatable. Furthermore, excessive daytime sleepiness (EDS) also has a negative effect on cognition (You et al., 2019). To the best of our knowledge, the exact relationship among cognition, the long-term rotating night SW (RNSW), and amount of daytime sleep after SW [regarding as daytime recharge (DTR)] in retired hospital nurses in China has been investigated in only a few studies. Therefore, in the present study, the effects of the long-term RNSW and DTR on cognition were investigated in the middle-aged subjects with ≥50 years.

## Materials and Methods

### Participants

**FIGURE 1:**
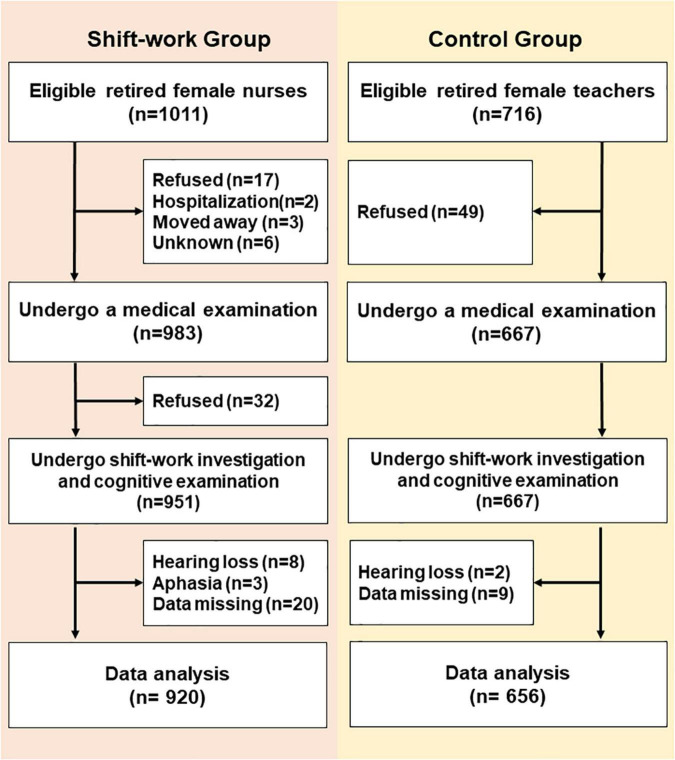
Flowchart.

A cross-sectional, comparable study was conducted in a medical examination center from September 4, 2019 to December 27, 2019, in Tianjin, China. A total of 920 retired nurses [age, (median) 63, (range) 50–91, years old] from five first-class tertiary hospitals and 656 retired female teachers [age, (median) 65, (range) 52–91, years old] from three senior middle school were analyzed (Figure 1). Based on the SW situation at the hospital, we defined RNSW group as participants worked at least once per week for 12 h at night (from 8:00 p.m. to 8:00 a.m. next day) for more than 1 year. Participants without a regular night SW were considered as the control group. The inclusion criteria were included as follows: women aged ≥50 years, Han nationality, educational level (≥12 years), and living in an urban area. The subjects who refused, moved away, were hospitalized, or were unable to complete the investigation due to hearing loss or aphasia were excluded.

This study was approved by the ethics committee at Tianjin Huanhu Hospital (ID: 2019-40). Informed consent was obtained from participants either directly or from her guardian.

### Study Procedure

#### General Interview and Cognitive Assessment

According to the centralized retired screening process, the participants completed the physical examination, blood tests (e.g., routine blood test, blood biochemical examination, thyroid function, syphilis, and vitamin B12 level), and a neuroimaging examination [either magnetic resonance imaging (MRI) or computed tomography (CT), selected by themselves]. After fulfilling the requirements, the participants who agreed to join in this study underwent the following steps.

The following demographic data were collected: age, sex (female), race (Han), educational level (≥12 years), marital status, living status, SW history [i.e., duration (years), the general number of night SW per month (times), and general DTR after night SW (hours)], lifestyle (i.e., habits of smoking and alcohol consumption), and medical histories, such as stroke, hypertension, diabetes mellitus (DM), hyperlipidemia, heart disease, and depression/anxiety. All participants were asked the following question: “Did you ever work night shift works?” If “Yes” means, the respondent was asked “How long do you sleep after each night shift work?” to reflect the DTR after night SW. All numbers required an integer that represented the highest frequency. The duration and frequency of RNSW were obtained access to the records of SW after getting the authorization (both participants and their hospitals).

Cognitive function was assessed using the Mini-Mental State Examination (MMSE) (Folstein et al., 1975), the Montreal Cognitive Assessment (MoCA) (Nasreddine et al., 2010), and the Activity of Daily Living Scale (ADL) (Bucks et al., 1996). The MMSE is a widely used cognitive screening test with scores ranging from 27 to 30 and is considered within the normal range (Arevalo-Rodriguez et al., 2015). A cutoff value of ≥26 for MoCA is considered normal cognition (Petersen, 2004). They were administered by qualified and experienced specialists in neurology, all of whom experienced the same training at Tianjin Huanhu Hospital in Tianjin, China. To ensure the accuracy of diagnosis, one neurologist and two neurology graduate students established the study protocol and performed a preliminary data review. A neurologist with expertise in dementia re-evaluated the data. The reliability for cognitive tests between the interviewers was required to exceed 85% (Trzepacz et al., 2015). For the interviewers who did not meet the standard, the neurologist re-evaluated the data for final diagnosis by revisiting the subjects on the following day.

#### Diagnostic Criteria for Cognitive Impairment

In the present study, CI [e.g., mild CI (MCI) and dementia] was diagnosed based on the criteria for MCI established according to published criteria (Petersen, 2004) and included all the following elements: (1) cognitive concern expressed by participants or caregivers; (2) CI in at least one domain, i.e., MMSE score of 24–26 points; (3) normal functional activities; and (4) no dementia and not taking medication for dementia (i.e., acetylcholinesterase inhibitors or memantine). The final MCI diagnosis was not based exclusively on score points but on all available data obtained. Subjects with MCI were classified into amnestic MCI (aMCI) or non-amnestic MCI (naMCI) (Petersen, 2004) and single domain or multiple domains based on the cognitive domain(s) with impairment, defined as 1.5 SD poorer than age-appropriate reference values (Gauthier et al., 2006).

Dementia was diagnosed according to the fifth edition of the *Diagnostic and Statistical Manual of Mental Disorders* (DSM-V) criteria (Joyce-Beaulieu and Sulkowski, 2016).

### Statistical Analysis

Based on the duration of RNSW, the participants were divided into three subgroups (i.e., 1–10 years, 11–20 years, and > 20 years), and each subgroup was subdivided into four categories (i.e., <1 h, 1–3 h, 3–5 h, and ≥5 h) based on the duration of DTR after RNSW.

Continuous variables were expressed as mean (±SD). Frequency distributions were used to analyze the qualitative variables. The differences in age, systolic blood pressure (SBP), diastolic blood pressure (DBP), heart rates (HRs), and scores of neuropsychological assessments (i.e., MMSE, MoCA, and ADL) were compared using the non-parametric test; and the χ^2^ tests between the control and SW groups were used to compare the difference of education, marital status, living status, lifestyle (i.e., habits of smoking and alcohol consumption), and medical histories, such as stroke, hypertension, DM, hyperlipidemia, heart disease, and depression/anxiety. Regression models were used to ascertain the associations among RNSW, DTR, and cognition.

The data were analyzed using SPSS version 25.0 (SPSS Inc., Chicago, IL, United States). A two-tailed *p*-value < 0.05 was considered statistically significant.

## Results

### The Demographic Data and the Clinical Characteristics of the Subjects

A total of 1,576 participants (920 in the RNSW and 656 in the control groups) were included in the study, and the basic characteristics are shown in Table 1. Participants with RNSW, whatever the duration might be, were more likely to be single (9.0%, *p* < 0.001) and living alone (10.6%, *p* = 0.001), and they also had a significantly higher frequency of hypertension (36.8 vs. 22.0%, *p* < 0.001) and hyperlipidemia (26.8 vs. 14.0%, *p* < 0.001), and lower scores of MMSE (27.1 ± 2.9 vs. 27.5 ± 3.4, *p* < 0.001), MoCA (23.2 ± 4.0 vs. 23.9 ± 4.1, *p* < 0.001), and ADL (20.6 ± 3.5 vs. 20.8 ± 3.9, *p* < 0.001) than participants in the control group.

**TABLE 1:** Participant characteristics.

Characteristics	Control	Duration of rotating shift work			
		All	1–10 years	11–20 years	>20 years
Num. of participants	656	920	205	383	332
Age, mean (± SD)	65.0 ± 5.2	65.4 ± 8.0	64.9 ± 7.3	65.0 ± 8.0^†^	66.2 ± 8.4
**Education, years, n (%)**					
12–15	456 (69.5%)	724 (78.7%)^‡^	136 (66.3%)	300 (78.3%)^†^	288 (86.7%)^‡^
≥16	200 (30.5%)	196 (21.3%)^‡^	69 (33.7%)	83 (21.7%)^†^	44 (13.3%)^‡^
**Marital status, n (%)**					
Married	528 (80.5%)	656 (71.3%)^‡^	148 (72.2%)^†^	279 (72.8%)^†^	229 (69.0%)^‡^
Single	2 (0.3%)	83 (9.0%)^‡^	22 (10.7%)^‡^	36 (9.4%)^‡^	25 (7.5%)^‡^
Divorced	11 (1.7%)	34 (3.7%)	6 (2.9%)	11 (2.9%)	17 (5.1%)^†^
Widow	115 (17.5%)	147 (16.0%)	29 (14.1%)	57 (14.9%)	61 (18.4%)
**Living status, n (%)**					
With spouse	500 (76.2%)	698 (75.9%)	161 (78.5%)	290 (75.7%)	247 (74.4%)
With others	118 (18.0%)	124 (13.5%)^†^	23 (11.2%)^†^	49 (12.8%)^†^	52 (15.7%)
Alone	38 (5.8%)	98 (10.6%)^†^	21 (10.3%)^†^	44 (11.5%)^†^	33 (9.9%)^†^
Smoking, yes, n (%)	28 (4.3%)	18 (2.0%)^†^	4 (2.0%)	6 (1.6%)^†^	8 (2.4%)
Alcohol consumption, yes, n (%)	19 (2.9%)	28 (3.0%)	7 (3.4%)	11 (2.9%)	10 (3.0%)
**Comorbidities, yes, n (%)**					
Hypertension	144 (22.0%)	339 (36.8%)^‡^	66 (32.2%)^†^	131 (34.2%)^‡^	142 (42.8%)^‡^
Hyperlipidemia	92 (14.0%)	247 (26.8%)^‡^	63 (30.7%)^‡^	105 (27.4%)^‡^	79 (23.8%)^‡^
DM	79 (12.0%)	125 (13.6%)	21 (10.2%)	55 (14.4%)	49 (14.8%)
Stroke	55 (8.4%)	89 (9.7%)	20 (9.8%)	31 (8.1%)	38 (11.4%)
Heart disease	37 (5.6%)	62 (6.7%)	11 (5.4%)	24 (6.3%)	27 (8.1%)
Anxiety/Depression	47 (7.2%)	77 (8.4%)	9 (4.4%)	32 (8.4%)	36 (10.8%)^†^
**Evaluation, mean (±SD)**					
SBP (mmHg)	135.6 ± 15.3	131.3 ± 18.8^‡^	128.3 ± 18.0^‡^	130.1 ± 18.1^‡^	134.7 ± 19.5
DBP (mmHg)	79.0 ± 7.2	78.7 ± 10.3^†^	78.9 ± 10.6	78.8 ± 10.4^†^	78.5 ± 9.9
HR (bpm)	72.5 ± 8.9	74.7 ± 10.9^†^	74.5 ± 10.6	75.0 ± 10.2^†^	74.3 ± 11.9
MMSE	27.5 ± 3.4	27.1 ± 2.9^‡^	28.0 ± 1.6	27.0 ± 3.4^‡^	26.9 ± 2.8^‡^
MoCA	23.9 ± 4.1	23.2 ± 4.0^‡^	23.7 ± 3.3	23.1 ± 4.3^†^	22.9 ± 4.0^‡^
ADL	20.8 ± 3.9	20.6 ± 3.4^‡^	20.2 ± 1.0^‡^	20.8 ± 4.4^†^	20.6 ± 3.2^‡^

*When compared with the control group, the significant value means ^†^p < 0.05 and ^‡^p < 0.001. DM, diabetes mellitus; SBP, systolic blood pressure; DBP, diastolic blood pressure; HR, heart rate; bpm, beats per minute; MMSE, Mini-Mental State Examination; MoCA, Montreal Cognitive Assessment; ADL, Activity of Daily Living Scale.*

### Associations Between Rotating Night SW and Cognition

**FIGURE 2:**
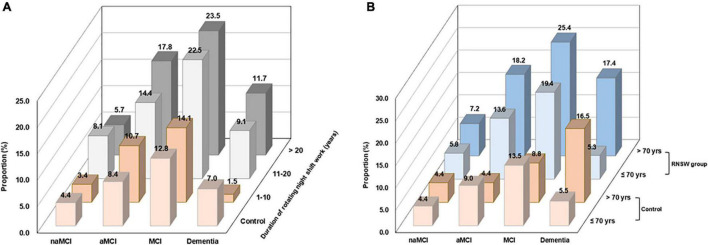
The proportions of CI by the duration of RNSW **(A)** and age **(B)**. CI, cognitive impairment; MCI, mild cognitive impairment; aMCI, amnestic mild cognitive impairment; naMCI, non-amnestic mild cognitive impairment; yrs, years old; RNSW, rotating night shift work.

Participants with RNSW showed a significantly higher proportion of MCI, both aMCI (14.4% in 11–20 years, *p* < 0.05, and 17.8% in >20 years subgroup, *p* < 0.001 vs. 8.4% in control) and naMCI (8.1% in 11–20 years vs. 4.4% in control, *p* < 0.05), as well as dementia (1.5% in 1–10 years, and 11.7% in >20 years subgroup vs. 7.0% in control, *p* < 0.05) than controls (Figure 2A). Moreover, participants aged >70 years in the RNSW group had the highest proportion of MCI than control, as shown in Figure 2B (25.4 vs. 8.8%, *p* < 0.05, and 13.5%, *p* < 0.001 in control), in particular, aMCI (18.2 vs. 4.4%, *p* < 0.001, and 9.0%, *p* < 0.05 in control).

Logistic regression analysis with two models is given in Table 2. Participants with > 20 years of RNSW could significantly increase the risk of CI (odds ratio [OR]: 2.00, 95% confidence interval: 1.47–2.72, *p* < 0.001), MCI (OR: 2.07, 95% confidence interval: 1.45–2.97, *p* < 0.001), in particular, aMCI (OR: 2.46, 95% confidence interval: 1.62–3.73, *p* < 0.001), and dementia (OR: 1.83, 95% confidence interval: 1.13–2.99, *p* < 0.05) than controls after adjusting for all factors. In addition, the same results were observed for participants regarding CI group and MCI (i.e., aMCI and naMCI) in 11–20 years of RNSW group.

**TABLE 2:** Logistic regressions for duration of rotating night shift work and cognition (OR and 95% confidence interval).

Parameter		Cognitive impairment		MCI		aMCI		naMCI		Dementia	
		Crude	Adjusted	Crude	Adjusted	Crude	Adjusted	Crude	Adjusted	Crude	Adjusted
Duration of rotating night shift work (years)	**Control**	1	1	1	1	1	1	1	1	1	1
	1–10	0.75 (0.49–1.14)	0.71 (0.46–1.09)	1.05 (0.67–1.66)	0.97 (0.61–1.56)	1.22 (0.72–2.05)	1.05 (0.61–1.80)	0.73 (0.32–1.71)	0.77 (0.32–1.83)	0.20 (0.06–0.65) †	0.20 (0.06–0.65) †
	11–20	1.87 (1.40–2.49)^‡^	1.77 (1.31–2.40)^‡^	2.06 (1.47–2.87)^‡^	1.92 (1.36–2.72)^‡^	2.01 (1.34–3.00)^†^	1.83 (1.20–2.78)^†^	2.15 (1.27–3.64)^†^	2.11 (1.21–3.68) ^†^	1.53 (0.96–2.43)	1.50 (0.91–2.45)
	>20	2.20 (1.64–2.96)^‡^	2.00 (1.47–2.72) ^‡^	2.27 (1.61–3.21)^‡^	2.07 (1.45–2.97)^‡^	2.62 (1.76–3.92)^‡^	2.46 (1.62–3.73)^‡^	1.60 (0.88–2.92)	1.34 (0.71–2.51)	2.07 (1.32–3.27)^†^	1.83 (1.13–2.99)^†^
		
Marital status	Married		1		1		1		1		1
	Single		1.17 (0.69–1.98)		0.95 (0.52–1.74)		0.76 (0.36–1.61)		1.49 (0.59–3.78)		1.81 (0.80–4.13)
	Divorced		1.36 (0.67–2.77)		1.29 (0.59–2.81)		1.68 (0.73–3.91)		0.38 (0.05–3.02)		1.41 (0.40–5.04)
	Widow		2.12 (1.26–3.58)^†^		1.53 (0.83–2.82)		1.28 (0.63–2.59)		2.69 (0.86–8.42)		3.98 (1.67–9.53)^†^
		
Living status	With spouse		1		1		1		1		1
	With others		0.83 (0.50–1.35)		0.82 (0.59–2.13)		0.80 (0.41–1.55)		0.71 (0.25–2.04)		0.78 (0.33–1.82)
	Alone		0.88 (0.50–1.55)		1.12 (0.59–2.13)		1.45 (0.72–2.94)		0.41 (0.11–1.55)		0.53 (0.20–1.40)
		
Stroke			0.97 (0.61–1.53)		0.83 (0.48–1.42)		0.94 (0.50–1.76)		0.65 (0.26–1.65)		1.26 (0.62–2.54)
		
Hypertension			1.41 (1.07–1.86)^†^		1.36 (0.99–1.87)		1.17 (0.80–1.70)		1.88 (1.11–3.18)^†^		1.52 (0.95–2.42)
		
Hyperlipidemia			1.16 (0.85–1.57)		1.23 (0.87–1.73)		1.53 (1.04–2.25)^†^		0.69 (0.37–1.30)		0.97 (0.58–1.63)
		
DM			1.50 (1.03–2.19)^†^		1.25 (0.81–1.94)		1.08 (0.64–1.81)		1.87 (0.90–3.88)		2.06 (1.13–3.74)^†^
		
Heart disease			1.08 (0.67–1.74)		1.19 (0.70–2.03)		1.12 (0.60–2.10)		1.24 (0.52–2.95)		0.91 (0.43–1.94)
		
Anxiety/Depression			0.85 (0.55–1.33)		0.88 (0.53–1.45)		0.22 (0.08–0.61)^†^		2.86 (1.58–5.18)^†^		0.85 (0.41–1.78)

*OR, odds ratios; MCI, mild cognitive impairment; aMCI, amnestic mild cognitive impairment; naMCI, non-amnestic mild cognitive impairment; DM, diabetes mellitus. Adjusted model: adjusted for marital status, living status, stroke, hypertension, hyperlipidemia, diabetes mellitus, heart disease, and anxiety/depression. When compared with the control group, the significant value means ^†^p < 0.05 and ^‡^p < 0.001.*

**FIGURE 3:**
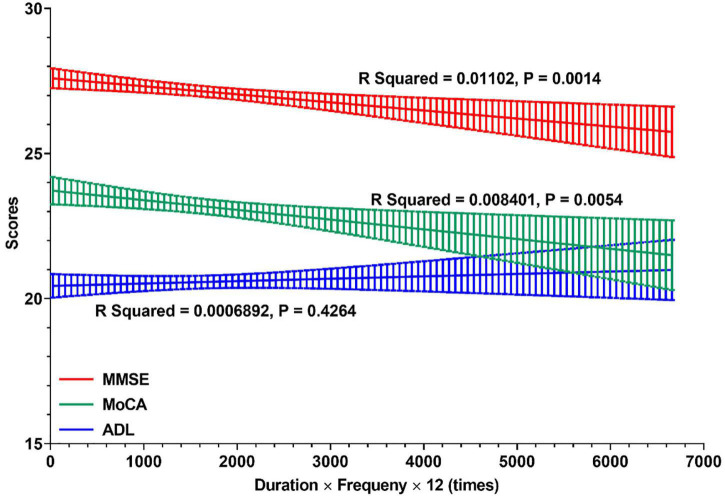
The relationships between cognitive profile and general times of night shift work. In this figure, linear regressions were used to display the relationships between cognitive profile (MMSE, MoCA, and ADL) and general times of night shift work in careers of nurses. And the general times of night shift work (X-Aris, times) = duration of rotating night shift work (years) × frequency (times per month) × 12 (month per year). MMSE, Mini-Mental State Examination; MoCA, Montreal Cognitive Assessment; ADL, Activity of Daily Living Scale.

Furthermore, the relationships between cognitive profile and general times of night SW in the career of nurses are shown in Figure 3. In all retired nurses with RNSW, there were significant negative relationships between general times and scores of MMSE (*R* squared = 0.01, *p* = 0.0014) and MoCA (*R* squared = 0.01, *p* = 0.0054). However, in the subgroups of general times with ADL scores, these variables were positively correlated, albeit not significantly (*p* = 0.4264).

### Effects of Daytime Recharge After Rotating Night SW on Cognition

**FIGURE 4:**
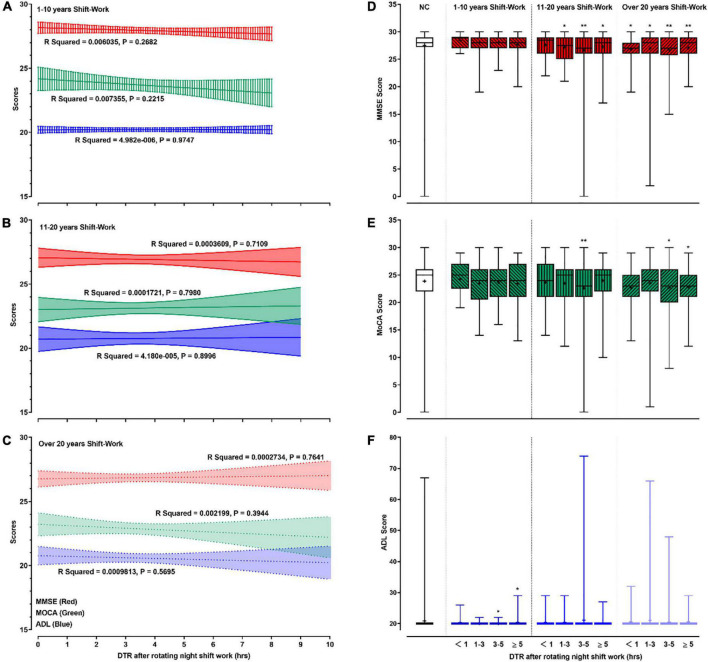
The relationships between cognitive profile and DTR. In this figure, panels **(A–C)** showed the linear regressions on cognitive profiles (scores of MMSE, MoCA, and ADL) and DTR after rotating night shift work each time. Panels **(D–F)** showed the mean (as “+”), median (middle line), and range (error bars) of cognitive profiles (scores of MMSE, MoCA, and ADL) in different subgroups by DTR plus duration of rotating night shift work. “*” and “**” in panels **(D–F)** were defined as *p* < 0.05 and *p* < 0.001, respectively when comparing with the control group. DTR, daytime recharge; MMSE, Mini-Mental State Examination; MoCA, Montreal Cognitive Assessment; ADL, Activity of Daily Living Scale.

The effects of DTR after RNSW on cognition are shown in Figure 4. Although we did not find significant differences between DTR after RNSW and the scores of MMSE, MoCA, and ADL by linear regressions (Figures 4A–C), the scores of MMSE and MoCA were significantly lower in 11–20 and > 20 years of SW groups, regardless of hours of DTR (Figures 4D,E).

Participants with 1–3 h, 3–5 h, and ≥5 h of DTR had higher proportions of CI (30.6, 33.3, and 30.0% in 11–20 years of night SW group; and 31.9, 38.6, and 30.3% in >20 years of night SW group, respectively) than participants in the control group (Table 3). In addition, MCI, including aMCI and naMCI, and dementia were also more significantly frequent among participants with different DTRs after 11–20 or >20 years of RNSW.

**TABLE 3:** The proportions of cognitive impairment by DTR and duration of rotating night shift work.

Duration of rotating night shift work (years)	DTR (h)	Cognitive impairment		MCI		aMCI		naMCI		Dementia	
		Num., Pro. (%)		Num., Pro. (%)		Num., Pro. (%)		Num., Pro. (%)		Num., Pro. (%)	
Control	–	130	19.8%	84	12.8%	55	8.4%	29	4.4%	46	7.0%
1–10	<1	1	3.8%^†^	1	3.8%	1	3.8%	0	0.0%	0	0.0%
	1–3	4	12.1%	3	9.1%	1	3.0%	2	6.1%	1	3.0%
	3–5	17	18.3%	16	17.2%	12	12.9%	4	4.3%	1	1.1%^†^
	≥5	10	18.9%	9	17.0%	8	15.1%	1	1.9%	1	1.9%
11–20	<1	9	26.5%	6	17.6%	4	11.8%	2	5.9%	3	8.8%
	1–3	22	30.6%^†^	18	25.0%^†^	10	13.9%	8	11.1%^†^	4	5.6%
	3–5	69	33.3%^‡^	45	21.7%^†^	31	15.0%^†^	14	6.8%	24	11.6%^†^
	≥5	21	30.0%^†^	17	24.3%^†^	10	14.3%	7	10.0%^†^	4	5.7%
>20	<1	11	35.5%^†^	7	22.6%	5	16.1%	2	6.5%	4	12.9%
	1–3	22	31.9%^†^	16	23.2%^†^	11	15.9%^†^	5	7.2%	6	8.7%
	3–5	64	38.6%^‡^	40	24.1%^‡^	31	18.7%^‡^	9	5.4%	24	14.5%^†^
	≥5	20	30.3%^†^	15	22.7%^†^	12	18.2%^†^	3	4.5%	5	7.6%

*DTR, daytime recharge; MCI, mild cognitive impairment; aMCI, amnestic mild cognitive impairment; naMCI, non-amnestic mild cognitive impairment; Num., number of participants; Pro., proportion. When compared with the control group, the significant value means ^†^p < 0.05 and ^‡^p < 0.001.*

Logistic regressions reflecting the attributing effects of DTR after RNSW on cognition are shown in Table 4. Participants with ≥1 h of DTR and ≥11 years of RNSW subgroup were about 2-fold more likely to experience MCI compared with subjects in the control group, especially with 3–5 h (OR: 2.35; 95% confidence interval: 1.49–3.68, *p* < 0.001). Furthermore, participants with ≥1 h of DTR in >20 years of RNSW were 2.18- to 2.71-fold more likely to experience aMCI after adjusting for confounding factors.

**TABLE 4:** Attributing effects of DTR after rotating night shift work on cognition (OR with 95% confidence interval).

Duration of rotating night shift work (years)	DTR (h)	Cognitive impairment		MCI		aMCI		naMCI		Dementia	
		Crude	Adjusted	Crude	Adjusted	Crude	Adjusted	Crude	Adjusted	Crude	Adjusted
Control	–	1	1	1	1	1	1	1	1	1	1
1–10	<1	0.16 (0.02–1.21)	0.16 (0.02–1.20)	0.25 (0.03–1.87)	0.24 (0.03–1.82)	0.38 (0.05–2.88)	0.36 (0.05–2.73)	na	na	na	na
	1–3	0.56 (0.19–1.62)	0.55 (0.19–1.59)	0.65 (0.19–2.17)	0.64 (0.19–2.14)	0.33 (0.04–2.47)	0.31 (0.04–2.31)	1.25 (0.29–5.50)	1.30 (0.29–5.82)	0.39 (0.05–2.96)	0.38 (0.05–2.87)
	3–5	0.91 (0.52–1.58)	0.91 (0.51–1.62)	1.32 (0.73–2.37)	1.28 (0.70–2.36)	1.51 (0.77–2.95)	1.33 (0.66–2.68)	0.96 (0.33–2.79)	1.02 (0.33–3.15)	0.15 (0.02–1.11)	0.15 (0.02–1.15)
	≥(5	0.94 (0.46–1.92)	0.84 (0.40–1.74)	1.31 (0.62–2.79)	1.13 (0.52–2.44)	1.7 (0.80–3.98)	1.44 (0.63–3.28)	0.42 (0.06–3.17)	0.48 (0.06–3.63)	0.27 (0.04–1.98)	0.27 (0.04–2.03)
11–20	<1	1.46 (0.66–3.20)	1.47 (0.66–3.28)	1.50 (0.60–377)	1.43 (0.56–3.63)	1.53 (0.51–4.56)	1.47 (0.48–4.48)	1.45 (0.33–6.43)	1.41 (0.31–6.38)	1.37 (0.40–4.72)	1.49 (0.42–5.31)
	1–3	1.78 (1.04–3.05)^†^	1.68 (0.97–2.91)	2.25 (1.26–4.05)^†^	2.09 (1.15–3.79)^†^	1.91 (0.92–3.98)	1.68 (0.79–3.55)	2.90 (1.26–6.69)^†^	2.80 (1.18–6.64)^†^	0.92 (0.32–2.65)	0.92 (0.31–2.72)
	3–5	2.02 (1.43–2.86)^‡^	1.97 (1.36–2.84)^‡^	2.04 (1.36–3.07)^†^	1.97 (1.29–3.02)^†^	2.15 (1.33–3.47)^†^	2.00 (1.21–3.31)^†^	1.84 (0.95–3.58)	1.94 (0.95–3.96)	1.99 (1.17–3.37)^†^	1.95 (1.10–3.44)^†^
	≥5	1.73 (1.00–2.99)^†^	1.62 (0.92–2.84)	2.17 (1.20–3.95)^†^	1.97 (1.07–3.64)^†^	1.95 (0.94–4.07)	1.81 (0.85–3.86)	2.59 (1.08–6.22)^†^	2.33 (0.93–5.80)	0.93 (0.32–2.70)	0.99 (0.33–2.93)
>20	<1	2.23 (1.04–4.76)^†^	1.87 (0.85–4.11)	2.19 (0.90–5.34)	1.89 (0.76–4.68)	2.39 (0.86–6.62)	2.29 (0.80–6.53)	1.81 (0.40–8.14)	1.24 (0.26–5.89)	2.29 (0.75–6.97)	2.00 (0.61–6.51)
	1–3	1.89 (1.10–3.26)^†^	1.71 (0.98–2.98)	2.13 (1.16–3.93)^†^	1.91 (1.2–3.56)^†^	2.24 (1.10–4.57)^†^	2.18 (1.05–4.54)^†^	1.93 (0.71–5.22)	1.43 (0.51–4.00)	1.46 (0.59–3.60)	1.30 (0.51–3.31)
	3–5	2.54 (1.76–3.66)^‡^	2.43 (1.65–3.57)^‡^	2.46 (1.59–3.78)^‡^	2.35 (1.49–3.68)^‡^	2.91 (1.78–4.74)^‡^	2.71 (1.63–4.52)^‡^	1.60 (0.74–3.48)	1.51 (0.66–3.45)	2.69 (1.57–4.60)^‡^	2.53 (1.41–4.53)^†^
	≥(5	1.76 (1.01–3.08)^†^	1.56 (0.88–2.76)	2.04 (1.09–3.82)^†^	1.85 (0.98–3.50)	2.50 (1.25–4.99)^†^	2.37 (1.16–4.82)^†^	1.18 (0.35–4.03)	0.99 (0.28–3.48)	1.24 (0.47–3.28)	1.04 (0.38–2.84)

*DTR, daytime recharge; h, hours; OR, odds ratio; MCI, mild cognitive impairment; aMCI, amnestic mild cognitive impairment; naMCI, non-amnestic mild cognitive impairment. Adjusted model: adjusted for marital status, living status, stroke, hypertension, hyperlipidemia, diabetes mellitus, heart disease, and anxiety/depression. When compared with the control group, the significant value means ^†^p < 0.05 and ^‡^p < 0.001.*

## Discussion

Our findings suggested that the long-term RNSW independently increases the risk of CI in late life and could not be compensated by the DTR. The proportions of CI increased ranging from 3.8 to 38.6% by the duration of RNSW and DTR, especially MCI presenting the increasing trend from 3.8 to 25.0%.

### Comparison With Other Studies

Currently, a meta-analysis revealed that the prevalence rate of MCI is 15.4% in people aged >65 years, and 11.1% (95% confidence interval: 9.1–13.3%) in those aged 60–69 years in China (Deng et al., 2021). The retired nurses not only had a higher proportion of MCI than controls in this study, but they also showed a higher proportion of MCI than some population-based surveys, even they were younger (mean age: 65.4 ± 8.0 years). Thus, the adverse effects of SW on cognition should be paid more attention, especially to the MCI stage.

The detrimental effects of SW on cognition are well-documented in studies of shift workers, both within nursing and outside of nursing. Anesthesia residents, emergency physicians, and miners who engaged in the night shift demonstrated a greater deterioration in cognitive functions compared to day shift workers (Saricaoğlu et al., 2005; Machi et al., 2012; Leso et al., 2021b). The laboratory setting and Aβ-PET evaluation confirmed the increase of Aβ after sleep deprivation (Ooms et al., 2014; Shokri-Kojori et al., 2018).

In addition to these acute, short-term settings, the long-term effects induced by prolonged exposure of SW on cognition had been an extremely interesting topic. Sleep deprivation or prolonged wakefulness caused by SW can interfere with a physiological decrease in Aβ-42 in morning, so as to elevate the risk of CI (Ooms et al., 2014). Our results are consistent with previous findings from cohorts (Rouch et al., 2005; Marquié et al., 2015; Jørgensen et al., 2020), as well as with public health recommendations (Leso et al., 2021a). In this analysis, the negative associations were observed between the duration, frequency of RNSW, and cognitive profile. In line with our findings, memory performance tended to decrease as the duration of SW increased. Immediate free recall was much lower in the group of 10–20 years compared to 1–4 years of exposure (Rouch et al., 2005). Workers with >10 years of RNSW had poorer cognitive scores than those with 1–10 years or never exposed (Marquié et al., 2015). Although there was no clear investigation on the relationship between the frequency of RNSW and cognition, a situation of “the frequency of night SW increases, so does the risk of incidences in a nurse’s work along with decreased alertness” had been mentioned by Borges and Fischer (2003). Even the risk of incidents appears to increase over successive shifts relative to the first night, for example, 6% during the second night, 17% during the third night, and 36% during the fourth night (Folkard and Tucker, 2003). However, different opinions pointed out no difference between the incidence of CI, even any of the cognitive domains, among those working with and without SWs (Rhéaume and Mullen, 2018; Weinmann et al., 2018; Thomas et al., 2019, 2021). The finding evidence of PET-CT also confirmed that the long-term SW was not associated with elevated brain Aβ levels nor with cognitive decline (Thomas et al., 2020).

Previous evidence on the cognitive effect of DTR in conjunction with RNSW is sparse, and there is no literature on the associations between the long-term SW and MCI, especially aMCI. In this study, we found that the long-term and high-frequency RNSW was related to MCI, especially aMCI, and dementia. The higher risks for CI, aMCI, and dementia were among women with at least 10 years of RNSW and at least 1 h of DTR, and the higher risks were among women with at least 20 years of RNSW and at least 3 h of DTR, compared with women who had no history of RNSW.

Although human studies show that EDS and the long-term exposure of SW can independently increase the risk of CI or all-cause dementia, there are few studies on DTR after SW (i.e., the combination of night SW and excessive DTR), and there is a lack of relevant objective research evidence. We suspect that the cognitive damage from excessive DTR after night SW may be doubled, meaning that it is a combination mechanism of sleep deprivation and EDS. Notably, studies based on neuropsychological assessment have found that the immediate and delayed recall is correlated with the duration and frequency of SW (Rouch et al., 2005; Marquié et al., 2015; Titova et al., 2016; Weinmann et al., 2018), while there is no difference in memory decline between those who left SW for at least 4 years and non-shift workers (Marquié et al., 2015; Titova et al., 2016; Thomas et al., 2020), which suggests that the effect of SW on cognitive function is reversible like previous reports among young adults (Boukhris et al., 2020). This abnormality may be associated with their work habits (e.g., banking sleep; Axelsson and Vyazovskiy, 2015) or high adaptation for SW, which decreased the length of DTR after SW but reduced the risk of cognitive decline.

### Potential Mechanisms

Taking into consideration that SW has a wide-ranging impact on circadian and sleep functioning, it seems likely that SW increases the risk of a general sleep disturbance, spread out over a multitude of comorbid sleep disorders. Hence, the RNSW is likely to share several possible mechanisms like sleep disorders involved in increasing the risk of CI. For example, RNSW and circadian rhythms play important roles in neurotransmitter metabolic function, such as leptin, ghrelin, thyrotropin, insulin, and melatonin (Sharma et al., 2017; Wei et al., 2020). Accumulating evidence also suggested that the association between SW and CI is consistent with the proposed mechanisms linking insufficient sleep to greater Aβ burden (Jørgensen et al., 2020; Thomas et al., 2020), which was considered as the onset of CI and served as an early biomarker of AD (Nassan and Videnovic, 2021). Sleep disruption, especially the reduction in slow wave sleep (SWS) and/or increased wakefulness, may suppress the function of the glymphatic system (Hablitz et al., 2020) that could result in the inopportune release and decreased clearance of pathogenic proteins, such as Aβ, which in turn may result in Aβ accumulation and the development of the symptoms of AD (Xie et al., 2013; Musiek and Holtzman, 2016). Furthermore, PET studies have proved that short sleep duration was associated with greater Aβ burden in cortical areas and the precuneus (Spira et al., 2013), and overlapped with the hypometabolism and atrophy regions in AD (Lehmann et al., 2013; Ingala et al., 2021).

Recent studies have found that sleep loss increases the permeability of the blood-brain barrier (BBB) through a variety of mechanisms, including increased inflammatory signaling and downregulation of tight-junction proteins (Rochfort and Cummins, 2015). The breakdown of the BBB impaired Aβclearance from the brain (Iliff et al., 2012). Endocytosis across the glia of the BBB is a newly appreciated function for sleep (Artiushin et al., 2018). Endocytic trafficking across the BBB is enhanced during sleep. Disruption of sleep and breakdown of the BBB affect endocytosis and impede the removal of toxic substances, whereas sleep/wake state affects endocytosis and the levels of endocytosis across subperineurial glia of the BBB also regulate sleep.

In addition, blocking the activity of endocytic trafficking across the BBB increases sleep and enhances sleep needs (Artiushin et al., 2018). Increasing evidence had underlined the role of EDS in the development of CI (Leng et al., 2019). Recent work led by Carvalho provides initial evidence that daytime sleepiness is associated with having less gray matter (Carvalho et al., 2017) and more Aβ accumulation over time (Carvalho et al., 2018) in the brain.

Gut microbiota and microbial metabolites play important roles in the development of AD (Doifode et al., 2021), and changes in gut microbiota have been proposed as a potential pathway linking SW and metabolic diseases because sleep loss and circadian misalignment could disrupt the intestinal microbiota (Reynolds et al., 2017).

Evidence has suggested that SW may also affect long-term health and safety (Kecklund and Axelsson, 2016; Vetter et al., 2016; Lizama et al., 2017; Shan et al., 2018). Consistent with previous studies, higher proportions of hypertension and hyperlipidemia existed in the RNSW group than controls. Changes in autonomic nerve control (Carter et al., 2018), increased oxidative stress, and acceleration of atherosclerosis (Javaheri et al., 2017) caused by sleep disorders are responsible for this consequence. Another interesting finding was that nurses were more likely to be single or divorced and lived alone after retirement. There is no research evidence for this, but we suspect that the instability of SW and unstable family life exacerbate the phenomenon. Participants with >20 years of RNSW had a higher proportion of anxiety/depression than controls, which may contribute to a higher divorce rate. In addition, evidence showed that unmarried status and mental disorders (e.g., anxiety and depression) could independently increase the risks of MCI and dementia (Helmer et al., 1999; Chen et al., 2021), while their interaction with CI is still worth exploring.

The major strengths of this study include the comparable design, large sample size, and poor recall or misperception bias by using the records of SW. And the relative homogeneity of this study population in educational attainment enhances the internal validity of our findings. To the best of our knowledge, this is the first study to investigate the associations among RNSW, DTR, and the risk of CI. Furthermore, participants with a high educational level were more likely to cooperate in cognitive test assessments. This study also has certain limitations. The participants were all Chinese Han female nurses with a high educational level, which limits the generalizability of our findings to other populations, particularly men and other educational, racial, or ethnic groups. Meanwhile, SW patterns can differ between professions and countries and are differentially associated with health. Because information on DTR was self-reported, potential exists for exposure misclassification. In addition, this study also lacks investigation on sleep quality and sleep disorders of the participants after retirement, and additional population-based epidemiologic studies or prospective cohorts that include wearable devices to record objective sleep time are needed to confirm the present study results.

## Conclusion

The long-term RNSW was associated with a higher risk of CI, especially aMCI and dementia, and the problem cannot be improved by DTR. The risk of CI among retired nurses with less than 10 years of SW and who had less than 1 h of sleep was significantly reduced, which indicated that managers should appropriately increase the SW flexibility, regulate the duration (no more than 10 years), and frequency of night SW. Though DTR after RNSW can help relieve fatigue, we encourage nurses to rest less than 1 h of DTR after night SW to weaken the adverse effects on cognition. Further studies are warranted to confirm our findings and clarify the underlying mechanisms.

## Data Availability Statement

The raw data supporting the conclusions of this article will be made available by the authors, without undue reservation.

## Ethics Statement

The studies involving human participants were reviewed and approved by the study was approved by the Ethics Committee at Tianjin Huanhu Hospital (ID: 2019-40). The patients/participants provided their written informed consent to participate in this study.

## Author Contributions

YJ designed this study. JG wrote the report. FW, X-DW, and ZC conducted the statistical analyses. JY, X-DW, FW, YY, LF, SL, PJ, JX, JZ, and ZS contributed to the interpretation and discussion of results and reviewed this manuscript. The collaborating authors contributed to the collection of the clinical data. All authors and the collaborating authors contributed to this article and approved the submitted version.

## Conflict of Interest

The authors declare that the research was conducted in the absence of any commercial or financial relationships that could be construed as a potential conflict of interest.

## Publisher’s Note

All claims expressed in this article are solely those of the authors and do not necessarily represent those of their affiliated organizations, or those of the publisher, the editors and the reviewers. Any product that may be evaluated in this article, or claim that may be made by its manufacturer, is not guaranteed or endorsed by the publisher.
